# The Anesthetic and Asphyxiating Effects of Nitrous-Oxid Inhalation

**Published:** 1896-02

**Authors:** J. D. Thomas

**Affiliations:** Philadelphia, Pa.


ARTICLE V.

### THE ANESTHETIC AND ASPHYXIATING EFFECTS OE NITROUS-OXID INHALATION.

BY J. D. THOMAS, D. D. S., PHILADELPHIA, PA.

Read before the Pennsylvania State Dental Society, July 9, '95.

*Mr. President and Gentlemen*:—The past year having been the fiftieth anniversary of the discovery of the anesthetic effects of nitrous oxid, by Horace Wells, the subject has received a pretty thorough handling by gentlemen far better equipped for the purpose than your essayist, therefore, it is approached with no little misgiving upon his part. But there is one phase of the subject which even yet is not clearly understood or accepted, and it would seem, while the present interest exists, that it is an auspicuous time to endeavor to substantiate the correctness of certain theories of the physiological effects of nitrous-oxid inhalation which the facts seem to warrant, as well as to whom belongs the honor of the great discovery.

There have been two theories advanced by gentlemen well versed in the knowledge of the effects of anesthetics, and each theory is based upon exceedingly plausible reasoning for its acceptance; so that as yet there is no thoroughly established understanding of what should be accepted by unanimous consent as the true explanation of the physiological effects of nitrous oxid.

It will be the object of the present effort to endeavor to establish the fact that nitrous oxid possesses as true and specific anesthetic properties, independent of the apparent asphyxiating accompaniment, as any of the anesthetic agents now known.

You all know that the two theories alluded to (both of which have many indorsers) are, first, that nitrous oxid produces its peculiar effects by deoxidation of the blood, or by true asphyxia from the want of oxygen; and second, that the anesthetic effects of nitrous oxid are due to an inherent property of the gas, which is distinct from and independent of the asphyxial concomitant frequently observed in connection with its administration.

In the *Dental Cosmos* for May, 1893, Dr. H. C. Wood —than whom I know of no one better qualified to speak as a scientific authority—gave the results of a series of experiments upon dogs, by first administering pure nitrous oxid to the obliteration of the corneal reflex. Then the same with three per cent. and five per cent. of oxygen, and finally inducing mechanical asphyxia by means of complete stoppage of the oxygen supply. The results of these experiments with the different percentages of oxygen mixture show a variation in time required to produce insensibility to the corneal reflex from two minutes and fifty seconds to twenty-three minutes; but those with pure nitrous oxid and mechanical asphyxia varied from one minute and forty-five seconds to three minutes and twenty-seven seconds for nitrous oxid, and one minute and forty seconds to two minutes and thirty seconds for asphyxia. The close approximation in time between the latter two led Dr. Wood to the conclusion that the theory of deoxidation was the correct one; yet in nearly the same breath he admits being surprised at realizing the powerful effect upon himself of nitrous oxid with five per cent. of oxygen, after only two or three deep inhalations,—to which had he given due consideration, he would at least have been led to question whether asphyxia was the only effect produced by nitrous oxid.

More recently, Dr. W. W. Belcher—in a paper read before a Union Meeting of the Fifth, Sixth, Seventh and Eighth District Societies of New York, at Buffalo, on October 13, 1894—gives the results of his personal experience in some interesting experiments of respiring nitrous oxid pure, and then by rebreathing air to the exhaustion of its oxygen; and he reports a striking similarity in the effects and sensations of each, which leads him to the conclusion again that the gas is asphyxiating and should be used with caution, but, carefully administered, it is the safest and most agreeable agent in use for producing unconsciousness,—and yet, if its effects are produced by asphyxia alone, why should it be any safer or more agreeable than numbers of other agents which we know will produce unconsciousness from the same cause?

It seems to me that the phenomena which distinguish true anesthesia from asphyxia have not received full consideration, and that their distinctive features and effects are not well understood.

Anesthesia is usually regarded as a condition of artificial sleep; but there may be several ways of producing artificial sleep or unconsciousness, which would not be a state of anesthesia.

From an experience of considerably more than one hundred thousand personal administrations, there is recognized convincing proof that a subject in a state of anesthesia is physiologically in precisely the same state or condition as one in a like degree of intoxication from the effects of the different forms of alcohol, only one is produced by inhalation and the other by intoxication, unconsciousness, and, finally, if pushed to that extent, death as a result. The effects are produced and exhibited in process,—first, by sensory paralysis, beginning at the periphery; simultaneously the cerebrum is affected, but loss of upper function or consciousness does not take place until a considerable peripheral anesthesia has been produced. The motor nerves may yet retain their power, without the guidance of volition. Then the motor nerves, and finally the medulla, succumb, when respiration and cardiac force cease. The order of the progressive effects is precisely the same in each.

We have from the different forms of opium poisons, effects exhibited in stupor and unconsciousness, and, finally, death; but no stimulation or intoxication. Similarly, we have like effects produced by the inhalation of poisonous gases, such as the fumes of sulfurous acid, mephitic gases, and carbonic acid gas. These are narcotic poisons producing their similar effects, one by alimentation, the other by inhalation,—the latter carrying with them a degree of asphyxia; but the final results cannot be ascribed alone to the want of oxidation.

Asphyxia produced by the inert gases, nitrogen or hydrogen, or by mechanical obstruction of the air-passages, results in unconsciousness, violent respiratory effort, stertor, muscular twitching, and sometimes convulsions, with death as a final result from lack of oxidation in the circulation. Recovery from the unconscious period being attended by extreme lassitude, and perhaps complete prostration.

While it is true that the asphyxial concomitant is a feature of profound nitrous-oxid narcosis,—perhaps also of all anesthetic agents,—and it is likewise true that anesthesia is a concomitant of the asphyxial state, it must be borne in mind that the order of appearance of anesthesia and asphyxial symptoms in the two states noted is widely different. As I have before stated, peripheral anesthesia is one of the earliest phenomena observed in nitrous-oxid inhalation, while in true asphyxia anesthesia is not produced until deoxidation of the blood has proceeded to a point of positive danger.

That nitrous oxid is an inert gas so far as its power to support life is concerned, is accepted to-day as a truth, there being no separation of the oxygen from the nitrogen at the temperature of the human body; so that it need cause no wonderment that many accept the theory of its producing its effects by the want of oxidation, for the breathing of pure nitrous oxid unmixed with oxygen or atmospheric air will in some cases produce all the symptoms of asphyxia, such as stertor, constriction of the glottis, muscular twitching, and even convulsions; but here the similarity ceases, for the effects pass away in a phenomenally short space of time, leaving the patient with no perceptible after effects of lassitude and prostration, except in very rare instances.

The length of time shown by Dr. Wood's experiments with asphyxia upon dogs, ranged from one minute and forty seconds to two minutes and fifteen seconds to produce annihilation of the corneal reflex. How long it may require to producet he same effect in man is of course problematical, but in a former paper upon the subject I recorded that a man exhibiting at the Walnut Street Theater under the pseudonym of "The Man Fish," performed several feats such as eating and smoking under water in a glass tank for the duration of three minutes and nine seconds, without showing any of the recognized symptoms of asphyxia other than considerable pallor of countenance and blueness of lips.

I have recently taken a record in one hundred cases of the time required in my own practice to induce anesthesia, in which there was a judicious admission of air during the inhalation, and they were brought to the operating point of unconsciousness in from thirty-two to fifty-nine seconds. It is possible that, by the inhalation of an inert gas, the displacement of the oxygen in the lungs might produce unconsciousness in less time than by the voluntary suspension of breathing of this "man fish;" but comparing his time of three minutes and nine seconds and the one minute and forty seconds of Dr. Wood's experiment upon dogs for asphyxia, and the time—less than one minute—required by the inhalation of the gas in private practice, certainly shows that there were other influences at work than simply deprivation of oxygen; and it is just this lack of practical information which has led to the confusion in the theories alluded to. It is clearly shown that the gas possesses true anesthetic properties, by its exhibition of exhilaration, intoxication and unconsciousness; and it is equally true that it will cause symptoms of asphyxia, as shown by cyanotic appearance, stertor, muscular twitching, etc.; but the essential difference in the two states is evinced by the fact that the narcosis of nitrous oxid is capable of extension to the point of surgical anesthesia before asphyxial symptoms supervene; the period of inhalation being so short that the risk of injury from asphyxia is so infinitesimally small that it is hardly worth considering. This is shown by the fact that in this country alone, it is computed that at least nine or ten millions of people have inhaled the gas for oral and minor surgery at the hands of men of all grades of ability, with but three or four deaths resulting therefrom. That the gas is a thorough anesthetic independent of the asphyxial accompaniment, is still further proven that by a mixture of oxygen anesthesia can be perfectly produced with little or none of the symptoms of asphyxia so prevalent when the pure gas is given. So thoroughly are they imbued with this idea in Europe that they use it altogether, Dr. Hewitt going so far as to state that in his position "the administration of nitrous oxid free from oxygen is irrational and unscientific." He does not say that oxygen will in all cases prevent a small degree of cyanotic appearance, but that the condition is such that even a member of the patient's family could witness the exhibition without becoming in the least alarmed, and that the anesthesia produced is if anything more profound than with nitrous oxid alone.

The same results are obtained by the admission of a proper amount of air during the inhalation. Blueness is in many cases scarcely apparent, while stertor, muscular twitching, and pharyngeal constriction are entirely obviated. Then too, as further illustrating the fact that asphyxia is not the anesthetizing force of the gas, two or three breaths of air, after the discontinuance of the gas, will restore absolutely the natural color of the blood; yet notwithstanding this obliteration of asphyxial symptoms we still have an operating unconsciousness sufficiently prolonged for twenty to thirty seconds, showing conclusively the true anesthetic properties of the gas, and that the cyanotic appearance is only an accompaniment, the injurious effects of which are infinitesimal, and can be kept in complete subjection by combining oxygen or admitting atmospheric air. The latter method has its advantages over the oxygen, in that we can control each individual case by the proper admission of air, as occasion requires, thereby producing greater uniformity, while with the admixture of oxygen in bulk, the variation in time with the different degree of constitutional strength of the subject is sometimes annoying, and when applied by the duplex inhaler the frequent changes and manipulation of the oxygen supply are not reassuring to the patient. Again, comparing the similarity of effects of alcohol intoxication and anesthesia, we find many constitutions able to withstand the effects of an inordinate amount of whiskey, while others are easily affected thereby; the one being nearly impervious to the diluted article, the others incapable of resistance. The same powers of resistance are presented with the gas, or any anesthetic. This explains why the admixture of oxygen in bulk would vary so much in different individuals. By using the nostrils as a valve and having the lips as a guide, the degree of cyanosis can be controlled, the stertor and muscle twitching excluded, and complete anesthesia produced; the small degree of blueness being no disadvantage, and in the ordinary course of daily practice doing no harm. The fact of there having occurred three or four fatal cases admonishes us that there is a very small degree of danger attending, but unfortunately these were cases where conditions were not taken sufficiently into consideration, and where the possibilities of risk were not clearly recognized. The dangers are clearly from the production of asphyxia.

The cases requiring caution are the very anemic, the very full-blooded, the slow breather, and persons of low vital force from grippe or over-work. In the anemic, the paucity of red corpuscles is so great that the least deprivation of oxygen is felt in the heart's action, which may be depressed almost to the point of syncope at the expiration of the second breath. In such a case, in the event of death, it would be from almost instant heart failure, and the post-mortem would not show the effects of asphyxia in great discoloration of the blood and tissues, for the circulation would have ceased before such a condition could have been brought about, yet death would be legitimately from the want of oxidation. In the full-blooded, stertor and constriction of the glottis take place rapidly, and the difficulty of getting a sufficient amount of air in the lungs in time to reoxidize the blood might result in cessation of respiration, and finally death from asphyxia. A case of this kind would be more accidental than from the physiological effects, for if the tongue is pulled forward to open the air-passages before the respiratory effort ceases, recovery is certain. In the slow breather, danger of suspension of breathing is as great as heart-failure in the anemic. In the experience of your essayist there are just as many people in apparent good health with weak respiration as there are with weak heart-force; people who seem never to take a breath exccept with voluntary effort, and then not more than twelve or fourteen to the minute. The color of the lips would indicate that their blood is never fully supplied with oxygen, and they require the full amount of their capacity to keep them going, the same as the anemic do for their heart-action. In either case they are intolerant of an insufficient supply, so that suspension may occur early in the administration, the heart's action continuing from ten to fifteen minutes after. In the event of death in such a case, the post-mortem would undoubtedly show the results of asphyxia in the discolored blood and tissues. These cases have had much to do with the acceptance of the theory of asphyxia being the only effect of nitrous-oxid inhalation.

In the cases of low vitality, the dangers are less, but the results might be from prostration, the nervous system being too weak to throw off or rally from the effects of the anesthesia, independently of any depressing effect of the small degree of the accompanying asphyxia. Such a case is reported at the meeting in Buffalo; the lady, a professional nurse, who, like many of her profession, presented appearances of over-work, to which was added severe suffering from neuralgia, for which a resection of the inferior dental nerve had been advised after some months of suffering, loss of strength and health. In the mean time, the cause of her trouble was located in a third molar. On the day of the extraction, she had taken three-quarters of a grain of morphia; complete prostration followed the operation, the respiratory effort entirely suspended, pulse hardly perceptible, and had it not been for the continued use of the Fell apparatus for artificial respiration, the patient would no doubt have died. Cases where such results might be looked for were quite numerous during the grippe epidemic of 1891 and 1892, but whether from grippe or other enervating conditions, the results might be the same. In my own practice, two cases in this condition were advised against taking the gas; one died within twenty-four hours, and the other three days after leaving my office. Had I given the gas, even had they recovered at the time, death would no doubt have been ascribed to the effects of the gas.

Attention is called to these cases to illustrate the fact that some knowledge of conditions must be had, so that we can use discretion and avold the dangerous results, by first recognizing the subjects which are likely to be so affected, and second by recognizing the symptoms of danger as they approach.

From the foregoing and from practical experience, it would appear to point to the conclusion that the inhalation of nitrous oxid does produce perfectly legitimate anesthetic effects as exhibited by stimulation, intoxication and unconsciousness, and, there being no separation of the oxygen from the nitrogen at the temperature of the human body, it is practically an inert gas so far as oxygenating the blood is concerned, which will, when its administration is pushed far enough, develop the asphyxial condition, the evil effects of which can be rendered practically *nil* by the proper combination of oxygen or air. Inasmuch as the condition of true surgical anesthesia precedes that of asphyxia, the latter should never be produced; and when it is, can only be attributed to a lack of intelligent understanding of the physiological action of the gas.—*Dental Cosmos.*

